# Imagining the “open” university: Sharing scholarship to improve research and education

**DOI:** 10.1371/journal.pbio.1002614

**Published:** 2017-10-24

**Authors:** Erin C. McKiernan

**Affiliations:** Departamento de Física, Facultad de Ciencias, Universidad Nacional Autónoma de México, Mexico City, Mexico

## Abstract

Open scholarship, such as the sharing of articles, code, data, and educational resources, has the potential to improve university research and education as well as increase the impact universities can have beyond their own walls. To support this perspective, I present evidence from case studies, published literature, and personal experiences as a practicing open scholar. I describe some of the challenges inherent to practicing open scholarship and some of the tensions created by incompatibilities between institutional policies and personal practice. To address this, I propose several concrete actions universities could take to support open scholarship and outline ways in which such initiatives could benefit the public as well as institutions. Importantly, I do not think most of these actions would require new funding but rather a redistribution of existing funds and a rewriting of internal policies to better align with university missions of knowledge dissemination and societal impact.

## Abstract 2

This Perspective article argues that universities should take action to support open scholarship that benefits society and to return to their core missions of knowledge dissemination, community engagement, and public good.

## Introduction

Over the last few years, we have seen growth of grassroots movements to increase access to scholarly products, such as articles, code, data, and educational resources (e.g., [1–5]). We have also seen a rise in the number of government and private funders mandating open access and open data [6,7] and the emergence of the Open Research Funders Group (http://www.orfg.org). These initiatives have been key in raising awareness and acceptance of open scholarship. However, despite these advances, I believe we have hit a wall that is impeding widespread adoption. While increasing numbers of academics may ideologically support sharing their work, many are concerned with how these practices will affect their career prospects and advancement [8–13].

Academic institutions are one of the primary influencers affecting how faculty perceive open scholarship and how willing they are to engage in certain practices [8, 13, 14]. Faculty often cite a lack of institutional support for open access, especially in evaluations, as one reason they are reluctant to publish in these journals [11]. Moreover, faculty express fear that open scholarship practices, especially those that fall outside the traditionally rewarded research products, will not only not be rewarded but may even hurt their evaluations. For example, one respondent of a 2011 survey of medical faculty [15] wrote,

*To my knowledge, community-engaged scholarship is perhaps a liability in the promotion process, because it slows work down and may result in fewer publications. Publications, by the number, still reign supreme here*.

Faculty understandably pay attention to what institutions value and where evaluation committees place the most weight to decide where to invest the most personal effort. As a University of Idaho faculty member wrote in response to a 2013 survey [11],

*What will we value at tenure and promotion? That will be the predominant driver of what we as a university community do. If public outreach and measure of its effectiveness can be captured and it becomes highly valued—then maybe that’s what we’ll be doing instead*.

A 2015 survey in the United Kingdom found that academics are increasingly tailoring their scholarly production and publication decisions to fit institutional evaluation criteria [16]. Thus, I believe universities are in a unique position to support open scholarship and break through some of the barriers to widespread adoption. This support could come in many forms, including recognition of open access and open data in promotion and tenure evaluations, small grants to support the development of open educational resources, and redirecting existing funds from proprietary software to support creation and training in open source solutions. Simple actions could demonstrate that universities value sharing, thereby changing faculty behavior. Such support could, in turn, have benefits for institutions, such as increased funding, visibility, and recruiting power. Most importantly, the sharing of scholarly outputs could help universities meet their stated missions to create and disseminate knowledge for broader public good.

### What should universities consider “open scholarship”?

There is no one unanimously accepted definition of open scholarship; the debate continues as to what the minimum requirements and best practices are for different types of open content [17]. Some of the earliest and perhaps most well-accepted international open standards are the Budapest Open Access Initiative (2002) [18], the Bethesda Statement (2003) [19], and the Berlin Declaration (2003) [20]—all of which deal with open access to articles.

At the time these declarations were written, they were revolutionary, and their original language still guides open scholarship efforts today. However, research has rapidly changed over the last 10–15 years, and projects are now producing much more than just articles, including large amounts of data, different types of digital media, electronic notebooks, and complex software. In recent years, open science has emerged as an umbrella term to refer to open access, open data, open notebooks, open source, or any other aspect of our work as researchers that can be shared [21, 22]. International standards for these products have emerged, including the Open Source Definition (2007) [23] for openly licensed software and the Panton Principles for open data (2010) [24].

More recently, there has been recognition that “open science” may not be as inclusive a term as we might like [25], and some have opted instead to refer to “open research” to include disciplines like the humanities [26, 27]. I will use the even broader term “open scholarship” to encompass sharing of research and nonresearch products, such as those arising from educational and outreach activities [28, 29]. I see inclusivity as crucial to the success of open scholarship as a social movement. While open scholarship can encompass all of the aforementioned practices, academics do not have to engage in all of these to contribute. Openness can be considered a continuum of practices [6]. Researchers can start with simple actions, like self-archiving free copies of their articles, and work their way up to sharing code, data, or notebooks. Educators can begin by sharing electronic copies of their class notes and work their way up to the creation of open textbooks or interactive online materials. It is important we welcome people at whatever level of sharing with which they are comfortable.

For this to work, it is in turn important that universities have ways of recognizing diverse scholarly products and different types of sharing. But with all the different standards, how are universities to determine what counts as open scholarship? I propose that universities take guidance from perhaps the simplest and all-encompassing international standard, the Open Definition from Open Knowledge, which states, "Open means anyone can freely access, use, modify, and share for any purpose" [30]. This definition can be applied to any educational or research product, allowing universities to set a clear baseline. Colleges, schools, and departments could then set more specific standards to fit disciplinary needs.

### Open scholarship can transform research and education

A comprehensive discussion of the benefits of open scholarship is beyond the scope of this paper (see instead [6, 31, 32]). Here, I focus on just a few ways sharing can transform research and education, falling largely into the democratic (“equal access for all”) and pragmatic (“sharing improves research and education”) schools of thought [22]. In each section, I begin by outlining some of the democratic and pragmatic benefits of open scholarship, then describe how I see such practices also benefiting universities and fitting in well with institutional missions. While many of the societal benefits of open scholarship have sometimes been considered to be at odds with the interests of institutions, I argue there are several points of intersection at which what is good for the public may also be good for the university. In my opinion, many universities have drifted away from their stated missions of knowledge dissemination, community engagement, and public good. Open scholarship provides an opportunity for universities to return to these core values.

#### Creating inclusive knowledge societies

In 2010, the United Nations Educational, Scientific and Cultural Organization (UNESCO) committed to the creation of Inclusive Knowledge Societies [33]:

*In the past, information and knowledge have too often been the preserve of powerful social or economic groups. Inclusive Knowledge Societies are those in which everyone has access to the information that s/he needs and to the skills required to turn that information into knowledge that is of practical use in her/his life*.

**Fig 1:**
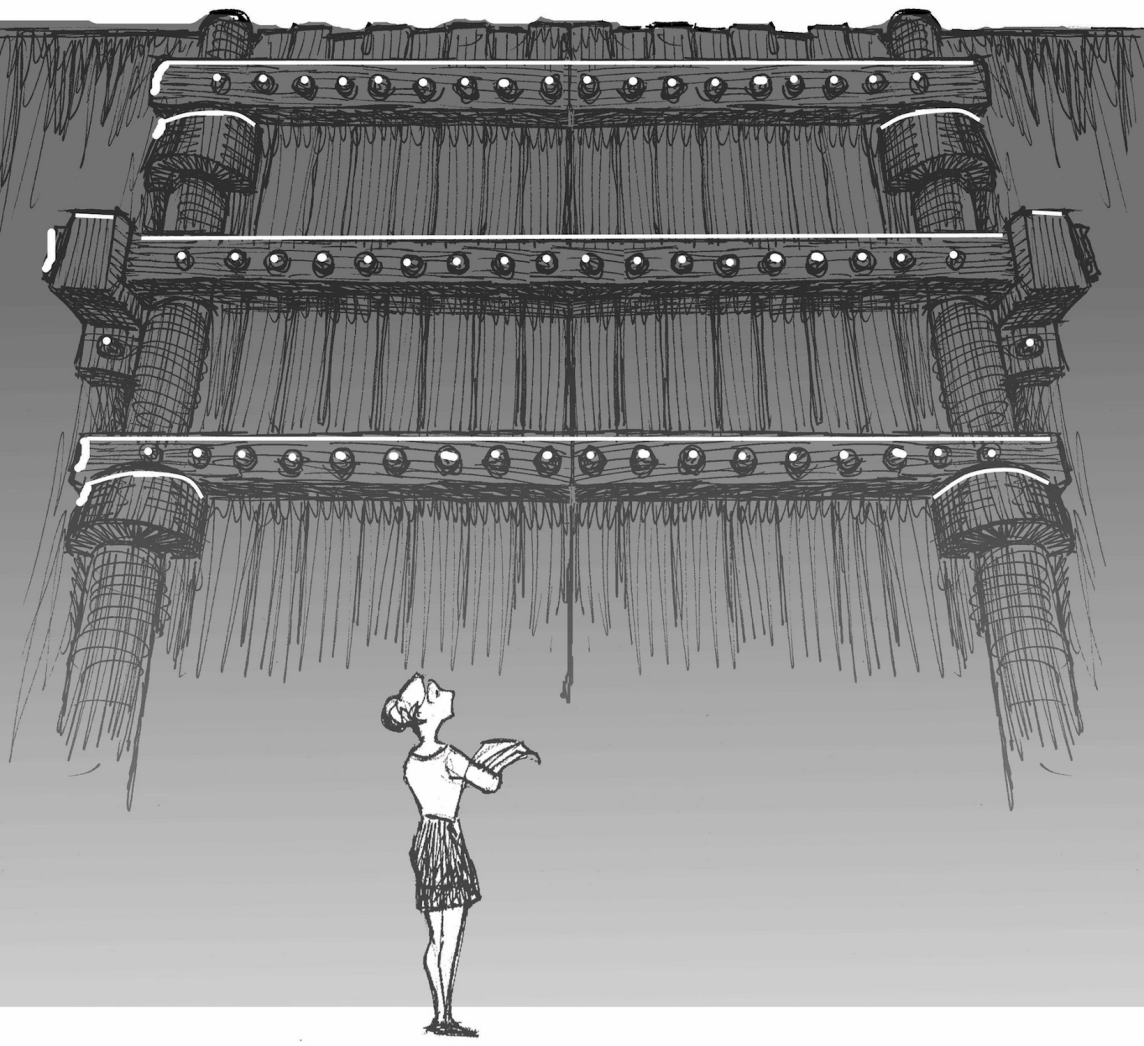
Scientific information is locked behind paywalls. People all over the world are locked out, unable to access information due to high subscription costs. *Image*: *John R*. *McKiernan and the “Why Open Research*?*” project* (http://whyopenresearch.org).

Currently, our societies are far from inclusive. All over the world, people lack access to scientific information (Fig 1). A study by Laakso and Björk reported that only 17% of 1.6 million articles published in 2011 were available without a subscription [34]. Studies up to 2012 [35] and 2015 [10] put the estimate around 22%–24%, although this number is likely to vary with discipline. A new study by Piwowar et al. estimates that, overall, 28% of the academic literature is free to access online, and although that number is growing, it was only 45% as of 2015 [36]. A study by the World Health Organization demonstrates the scope of the problem [37]:

*In the lowest-income countries, 56 percent of the institutions had no current subscriptions to international journals and 21 percent had an average of only two journal subscriptions. In the tier with the next-lowest incomes, 34 percent of institutions had no current subscriptions, and 34 percent had two to five journal subscriptions*.

Just recently, it was announced that scientists in Germany, Peru [38], and Taiwan are losing access to Elsevier journals, in part because of increasing subscription fees [39]. Rising costs have also made textbooks unaffordable, negatively impacting education [40, 41]. As Nicole Allen, Director of Open Education for the Scholarly Publishing and Academic Resources Coalition (SPARC), has said, "Students can’t learn from materials they can’t afford" [42]. A lack of access can impede learning and slow discoveries. Science itself could suffer, too, losing valuable perspectives when many researchers can't participate in their rapidly evolving fields.

Open scholarship democratizes access to information by making research available to all, regardless of financial resources—a necessary, though not sufficient, step in creating a true "knowledge democracy" [43]. Removing financial barriers helps those in low- and middle-income countries keep up to speed with their fields, potentially increasing their participation and the diversity of perspectives in research. (Improved access is a necessary condition but should not be seen as the magic bullet that will resolve all inequalities [44]. Much more than access to information is required to increase participation in research, including improved infrastructure and better funding for research in these countries [45]. These are not easy problems to solve, but they should not be ignored.) In addition, when research is open, participation is not limited to academics. The fast-growing area of citizen science is a testament to what can be achieved when we encourage contributions from outside the academy [46]. In sum, open scholarship allows us to create Inclusive Knowledge Societies [33], which I would argue should be one goal, if not the goal, of universities.

#### Open scholarship can make universities more inclusionary

Universities are by nature exclusionary—there are limited spots and often only those with the highest grades and test scores are accepted. In the 1940s, people began referring to academic institutions as ivory towers, where an elite few engaged in intellectual pursuits, largely "disengaged" from the concerns or needs of the public [47]. If anything, the perception of universities as ivory towers has only grown over the last decades, as competition for student and faculty positions increases, leaving many more on the outside. As Shapin writes, "Today, almost no one has anything good to say about the Ivory Tower and specifically about the university in its supposed Ivory Tower mode" [47].

How can institutions move away from this negative image and become more inclusionary? Increasing acceptance rates is not feasible for economic and infrastructure reasons. However, universities can allow everyone access to the knowledge created inside their walls. Open educational resources (OERs) are a prime example of openness increasing inclusion [48, 49] and are especially important for increasing access to education in developing countries [50, 51]. When universities make lecture notes, exams, and textbooks openly available online, even those who cannot attend in person can benefit from what the institution has to offer. In fact, 20%–50% of surveyed visitors to open courseware (OCW) websites identify as "self learners" [52]. Educators also benefit from OCW sites, making up around a quarter of visitors from regions like Latin America, Eastern Europe, and the Middle East and North Africa [53]. As an educator in Mexico, I use open textbooks available through projects like OpenStax (https://openstax.org), run by Rice University, because I know my students cannot afford expensive textbooks but still need access to quality information to learn.

The recent growth of massive online open courses (MOOCs) [54], particularly large-scale, free course initiatives by prestigious United States universities (e.g., edX, https://www.edx.org, run by Harvard and the Massachusetts Institute of Technology), is one indication that institutions are recognizing their exclusionary nature as a problem and trying to improve access to education by lowering financial and presential barriers. While this can be seen as positive, it is also important to not lose sight of the goal to increase inclusion. The issue is not just access but also participation [45]: who is creating knowledge, and how do their experiences influence and inherently bias educational content? If the majority of OERs are produced by prestigious US universities, it represents another form of exclusion and reinforces the problem of Western perspectives (and the English language) dominating educational content [44, 50, 52]. Resource-rich universities in Canada, the US, and Europe should look for ways to support, raise visibility, and increase the use of OERs from other countries with diverse global perspectives to facilitate a "true knowledge exchange" [44]. An example of an OER project from Africa is the Science Education Exchange for Sustainable Development (SeeSD; https://www.seesd.org), based in Senegal, which is designing open resources to improve access to education and participation in science, technology, engineering, and mathematics (STEM). SeeSD is also developing a MOOC-style online learning platform called Afreecademy (http://afreecademy.org). Examples from South Asia and Southeast Asia, respectively, include Sakshat from India (http://www.sakshat.ac.in) and the Vietnam Open Educational Resources program (http://www.voer.edu.vn). More on OER projects in Asia can be found in [55]. An example from Latin America comes from the Universidad Nacional Autónoma de México (UNAM), where I work. UNAM does not have a financial barrier to entry, because tuition is not charged, but there is a huge demand for a small number of places. UNAM annually accepts only approximately 10% of bachelor's degree applicants through open admissions testing [56]. In 2011, the university launched “Todo la UNAM en Línea” (“All of UNAM online”, http://www.unamenlinea.unam.mx) to provide open access to the knowledge generated by the institution for the benefit of society.

Beyond the societal benefits, universities have reasons to adopt OERs to benefit their own student population. Surveys show that many students do not buy textbooks due to high costs, and that this may be associated with failure to pass classes and high dropout rates [41, 57]. OERs can help address financial disparities among students and may improve performance. In 2013, Tidewater Community College became the first US institution to offer a degree program using exclusively OERs. Not only have they shown it is feasible to run such a program but, also, data up to 2015 indicate that switching to OERs is associated with better student learning outcomes and retention rates, which may ultimately lead to quicker graduation times [58]. Such statistics on student performance, retention, and degree completion contribute to university rankings and, consequently, to funding and recruitment power.

While there are benefits for students and the university, it should not be overlooked that the development of OERs implies investment of time and effort by faculty. In addition to content creation, there exist higher standards when materials are shared via public platforms. For example, the University of California, Berkeley, was recently told by the US Department of Justice that their online open educational materials did not meet accessibility standards required by the Americans with Disabilities Act [59]. There are additional concerns with OERs, such as ensuring that images pulled from primary sources are licensed for reuse. This added effort, in turn, requires institutional recognition and support if OER creation is to be undertaken by more than just a few altruistic individuals. Some evaluation systems for hiring, promotion, and tenure put less weight on the publication of books and book chapters than journal articles. Worse yet, electronic resources may not be recognized at all if not published by “prestigious” publishing houses [60]. OER creation must be recognized in its multiple forms if faculty are going to participate. A few steps universities could take to support OERs are listed in Box 1.

**Box 1. Supporting open educational resources (OERs) and practices.**

1. **Redirect textbook purchasing funds to support faculty.** Purchasing textbooks involves buying a limited number of copies and requires buying new editions every few years. Money would be better invested in openly licensed, electronic textbooks, for which there is no limit on copy number, and these e-books can be updated in real time as new discoveries are made. Faculty could be awarded small grants to write, maintain, or even peer review open e-books. Support could also include providing formal guidance on accessibility standards and licensing issues to lower the burden of OER creation for faculty.
2. **Develop 2–5-year plans to convert existing degree programs to OERs.** Plans of study typically undergo periodic evaluations. This would be a natural time to review class syllabi, search for open alternatives to current textbooks, and identify areas in which OERs are missing and could be developed by faculty.
3. **Require all new degree programs to use primarily OERs.** If new degree programs are proposed, faculty can design core courses to rely primarily on OERs from the start. Academic boards reviewing these proposals can be advised to evaluate OER use as part of the approval criteria.
4. **Devise incentives for OER creation and open educational practices.** One incentive would be positive mention of OERs in guidelines for promotion and tenure. An example of such a policy comes from the University of British Columbia, which lists creation of OERs as one way faculty can demonstrate "evidence of educational leadership" [61]. Another incentive could be teaching prizes based on open educational practices. This would be one way for institutions to establish prestige around open education and signal their support.

#### Sharing can increase the societal impact of university research

As part of their mission statements, many universities emphasize the importance of contributing to society through the “dissemination of knowledge.” For example, Cornell University’s mission [62] is as follows:

*Cornell's mission is to discover, preserve, and disseminate knowledge; produce creative work; and promote a culture of broad inquiry throughout and beyond the Cornell community. Cornell also aims, through public service, to enhance the lives and livelihoods of our students, the people of New York, and others around the world*.

These are excellent goals for a university. But how effectively is knowledge transmitted, and how can it benefit the community, if a large percentage of our society can't access it? Open scholarship can help universities fulfill their missions by sharing research outputs so they have the quickest and broadest societal impact.

Members of society want and need access to research. The “Who Needs Access?” project (https://whoneedsaccess.org) has documented stories from nurses, patients, teachers, and small business owners who tried to access scholarly articles for personal or professional uses but were unable. The Open Access Button project (https://openaccessbutton.org) has logged thousands of request for articles from nonacademics all over the world who do not have access. When articles are available, the public is eager to access them. A recent survey of users of Latin American open access platforms found that up to a quarter of respondents were from outside universities, including nonprofit, private, and public sector employees [63]. Around 50% of users were students, including many at the elementary and high school levels. The author points out, as follows, that these results have implications for how we measure impact in university evaluations:

*The alternative impact of research uncovered here [is] again evidence of the shortcomings of considering…a limited notion of the term “impact.” It makes little sense to use citations as the sole measure of evaluating research and researchers when over three quarter [sic] of the use of research is from non-citing publics*.

Likewise, open data can have impact far beyond university walls. Two projects—Open Data's Impact (http://odimpact.org) [64] and the Open Data Impact Map (http://opendataimpactmap.org)—are collecting case studies from all over the world to show how philanthropic, public health, social justice, and other similar organizations are using and sometimes also creating open data to improve society. For example, a quick search of Open Data Impact Map reveals nonprofit organizations in Mexico using open data to promote environmental protection and defense of indigenous lands (CartoCrítica, http://www.cartocritica.org.mx), improve Mexican economic competitiveness (El Instituto Mexicano para la Competitividad, http://imco.org.mx), and better the lives of Mexicans living with HIV (Derechohabientes Viviendo con VIH del Instituto Mexicano del Seguro Social, http://www.dvvimss.org.mx).

The potential for shared code to benefit society is only limited by what people can think to program. For example, the open source application REFUGE Restrooms (http://www.refugerestrooms.org) helps transgender, intersex, and gender nonconforming people find safe restrooms to use to avoid harassment and possible violence. HospitalRun (http://hospitalrun.io) is open source software that helps hospitals in low- and middle-income countries manage patient records. High Tech Humanitarians (http://www3.hthumanitarians.org), supported by the Institute of International Humanitarian Affairs at Fordham University, is a collaborative platform for people to share and improve open software and hardware tools for addressing societal issues like clean and renewable energy, distribution of medical resources, disaster management, and protection of human rights. Several of the projects on High Tech Humanitarians involve participation from universities like MIT and Harvard.

Academic institutions that share research products can be part of social change and improvement. The Earlham Institute in the UK is an example of a research institute that has committed to open scholarship, writing, "A determined commitment to open science, open access and open data allows us to have a significant impact" [65]. Earlham has published several "impact stories" (http://www.earlham.ac.uk/impact-stories) describing how open scholarship is aiding in their research efforts to improve the global food supply, protect animals and ecosystems, and create new technology. Having impact outside the academic environment reflects positively on a university and can increase its funding and recruitment power. Funders often ask for broader impact statements and may be more likely to award funding to researchers and institutions with a history of translating research into action. In addition, young students want to go where they see potential to effect change.

A university's societal impact depends on the commitment of faculty to transforming their research into reusable information, sharing, and participating in community outreach. As said before, if we want such commitment, universities must develop ways of recognizing and rewarding these activities. Traditional scholarly metrics, like the number of articles published and journal impact factor, give an incomplete picture of true impact. In my opinion, we need a broader perspective (see Box 2).

**Box 2. Recognizing nontraditional scholarly impact.**

1. **Recognize code and data in promotion and tenure evaluations.** Shared code and data should be recognized in academic evaluations as at least equal in value to published articles. Code and data citations can be measured but will likely underrepresent the use of these products, especially outside the academic sector. Additional metrics, such as repository follows, forks, pull requests, and other measures of community engagement should also be considered.
2. **Recognize, celebrate, and support outreach activities.** Many universities describe outreach as a core part of their missions but sometimes do little to support it in practice. Recognition could start with simple actions, like providing space on academic evaluation forms for faculty to describe how they are helping the university meet its commitments to the community through their outreach efforts. Celebrating these efforts could include circulating press releases or awarding faculty prizes for public engagement. If possible, cover expenses for faculty to take a day and visit local schools or clinics.
3. **Consider altmetrics as one measure of broader impact.** Nonprofit organizations, patient groups, and grassroots communities often use social media to share and communicate research of interest to them. Altmetrics provide measures of how widely scholarly products are being shared and discussed by groups who may be unlikely to formally cite work.
4. **Allow faculty to include narrative summaries of their impact.** Numbers alone will not capture the impact scholarly products have outside university walls. Faculty should be allowed to include descriptions of use cases in their annual reports or tenure packets, e.g., how their data was used by a local hospital or their software used by a local school. Universities could highlight interesting impact stories by publishing them on their website.

It is important to emphasize here that it will not be enough for universities to simply provide space for faculty to describe their outreach activities or public impact. If the university does not signal to the academic community that it values these things, they will likely continue to be largely ignored by evaluation committees in favor of more traditional scholarly products. If there are more university press releases about *Nature* or *Science* papers than school mentorship programs, for example, then prestige will continue to be defined by high-profile papers and not public engagement. The university can help redefine prestige; it can influence what becomes high profile in academic circles. As suggested in Box 2, celebrate outreach events with press releases, award faculty prizes for community engagement, and highlight public impact stories on the university website. Such actions signal to academics and the public that the university is truly committed to the ideals outlined in their mission statements.

#### Accelerating the pace of discovery

Sharing research allows for increased communication within and across disciplines and can encourage diverse approaches [66]. Sharing code and experimental protocols allows others to test and improve solutions. Sharing data allows others to perform new analyses, which could lead to new discoveries. To my knowledge, there have been no controlled studies comparing the pace of private versus public projects, but there are powerful anecdotal examples to support the idea that sharing can accelerate the pace of discovery.

The Human Genome Project (HGP) was one of the first high-profile projects to commit to open scholarship. In 1996, HGP researchers agreed to rapid data sharing [67]. This sharing accord, known as the Bermuda Principles, has been hailed as “revolutionary,” accelerating the huge task of sequencing billions of base pairs and leading to new gene discoveries [68].

In 2008, chemist Matthew Todd and colleagues began openly sharing their electronic laboratory notebooks as part of a research project to synthesize a drug to treat a parasitic disease [69]. The project attracted outside collaborators, and the suggestions made helped the researchers find a solution to their drug synthesis problem. Todd and coauthors write [69],

*…the research was accelerated by being open. Experts identified themselves, and spontaneously contributed based on what was being posted online. The research therefore inevitably proceeded faster than if we had attempted to contact people in our limited professional circle individually, in series*.

Todd now works as the lead researcher on the Open Source Malaria project, which openly shares all their electronic notebooks in real time to accelerate the search for malaria drugs [70].

In 2009, mathematician Tim Gowers launched the Polymath Project to experiment with open collaboration as a way to solve difficult math problems. Using a blog and a wiki to share ideas, "progress came far faster than anyone expected" [71]. Collaboration began on February 1, and by March 10, a solution was found. The project also shed light on the discovery process:

*For the first time one can see on full display a complete account of how a serious mathematical result was discovered. It shows vividly how ideas grow, change, improve and are discarded, and how advances in understanding may come not in a single giant leap, but through the aggregation and refinement of many smaller insights*.

In 2015 and 2016, in light of recent Ebola and Zika outbreaks, the World Health Organization [72] as well as funders and publishers [73] came out in support of data sharing and preprints to quickly disseminate information and accelerate responses to public health emergencies.

#### Accelerated discovery can give universities an edge

In 2016, acknowledging the potential for open approaches to accelerate discovery, the Montreal Neurological Institute (MNI), part of McGill University in Canada, announced its intention to become an open science institute [74]. Faculty at the institute have committed to sharing articles, code, data, and even physical samples and to not patent their research. In regards to not receiving patent income, the director of the institute, Guy Rouleau, says [75],

*Of course there is a risk that we might lose the economic returns of a blockbuster drug or a new intervention, but we are ethically committed to taking that risk, as the bigger risk is for our patients who are waiting for answers and new treatments*.

Rouleau says their support of open scholarship is already bringing in "highly talented researchers and trainees" [75]. This recruitment power may be seen by other universities that support open approaches, especially if these approaches lead to accelerated discoveries. When researchers are the first to make a discovery, it brings visibility and prestige both for the individuals and their institution, whose name is usually featured prominently in press releases and journal publications. This prestige, in turn, can benefit the university by attracting students and faculty as well as funding from public and private sources.

Participation in MNI's open scholarship initiative will be voluntary, and faculty can decide to independently patent their discoveries. However, MNI will not financially or administratively support faculty in doing so [74]. I think this sets an important precedent. The institution's approach is, “We will not force you to share your work, but we will not help you to lock it up.” This approach could be implemented by other universities, allowing faculty to retain academic freedom but making it clear where the institution stands on sharing. This and other ideas for supporting open collaboration and faster discovery are listed in Box 3.

**Box 3. Supporting open collaboration and accelerated discovery.**

1. **Remove financial and administrative support for patents.** As at the Montreal Neurological Institute (MNI), faculty could be allowed to patent but would not receive funds or help filing. Most patent offices operate at a deficit [76, 77], so this should not present significant income loss for many universities, and funds could be redirected.
2. **Redirect funds to hire grant and scholarly communication personnel.** Funders are increasingly awarding grants for open scholarship projects [6]. Having personnel dedicated to finding these opportunities and helping faculty submit applications could be profitable for the university. Hiring scholarly communication personnel to write research summaries or organize outreach could help universities raise visibility and find new partners.
3. **Organize academic “cross-pollination” events.** Many university events are targeted at single departments, with few opportunities for students and faculty from different disciplines to interact. Schedule events with broad interest and invite multiple departments. Scholarly communication personnel could be in charge of organization and diffusion.
4. **Establish shared, interdisciplinary laboratory spaces.** Laboratory space is at a premium and often, there are not enough resources for everyone. By pooling resources and establishing shared spaces co-run by researchers from different departments, one space can serve multiple uses, as well as foster interdisciplinary communication and projects. I co-run such a collaborative space at UNAM with professors from biology and mathematics.
5. **Develop ways to recognize collaborative efforts.** Collaboration is hard to measure and is discipline dependent. However, a place to start could be to ask faculty to submit short narratives of their collaborations, both inside and outside the university and within and across disciplines.

#### Addressing the reproducibility “crisis”

In recent years, large-scale projects in the fields of psychology [78] and cancer biology [79, 80] have attempted to reproduce key findings and found a low rate of reproducibility. These problems have become so prevalent that it has led many to say that science is facing a reproducibility crisis [81]. Last year, an article in *Nature* described work by researchers to reproduce 50 studies in cancer biology and the difficulties they faced obtaining original data [82]. In several cases, authors did not respond to requests for data. In another, data were only obtained after a year of trying. Many authors, while willing to participate, had trouble finding the original data, indicating poor data management.

We can only expect to reproduce a study if we know exactly what was done and how. Currently, too many crucial details remain hidden. Researchers struggle to recreate experimental methods using only details provided in original papers [83]. A 2015 study by Womack found that just 13% of articles in the top tier journals he examined shared their underlying data [84]. I believe the best way to improve reproducibility is to ensure that full experimental protocols, raw data, and analysis code are openly available and licensed for reuse.

Several researchers are leading the way in reproducibility [85–87]. In 2012, Lorena Barba, a professor at George Washington University, published the "Reproducibility PI Manifesto" describing her efforts to make the research in her lab more reproducible [85]. For Barba, this means (1) all code is under version control and shared publicly, (2) code undergoes "verification and validation" and reports are also shared, (3) data and scripts to recreate figures are openly licensed, (4) manuscripts are posted as open preprints, and (5) her lab's articles include a reproducibility statement. Barba also considers it her responsibility to teach her students about reproducibility. With respect to the learning involved, she writes [86],

*My students don’t resent investing their time in this. They know that practices like ours are crucial for the integrity of the scientific endeavor. They also appreciate that our approach will help them show potential future employers that they are careful, conscientious researchers*.

#### Reproducibility can affect university reputation

For universities, having "careful, conscientious researchers" [86] is to their benefit. When research is reproducible, it can reflect positively on the institution and their standards. For example, just recently, the Memorial Sloan Kettering Cancer Center received positive press in *Science* magazine when one of their researcher's leukemia studies was successfully reproduced by an independent group [88]. In contrast, when research is not reproducible or, even worse, is suspected to be fraudulent, this can reflect negatively on an institution. No institution wants the effort, expense, or publicity involved in investigating one of their researchers for fraud. Therefore, it is in the interest of universities to encourage researchers to be transparent and make their research more reproducible. How can universities accomplish this? See Box 4.

**Box 4. Increasing transparency and reproducibility.**

1. **Provide incentives for researchers to preregister their studies.** Registering hypotheses, data collection, and analysis plans before conducting research can diminish bias and decrease selective reporting [87]. The Center for Open Science offers a US$1,000 prize to researchers who preregister their studies [89]. Universities could provide small financial incentives to faculty. Evaluation committees could place more weight on preregistered projects.
2. **Encourage code and data sharing under version control.** Universities could let code and data sharing be voluntary but state that these products will only be counted in hiring, promotion, and tenure evaluations if they are shared in an open repository with version control, like GitHub or BitBucket.
3. **Recognize preprints as valuable research products.** Sharing preprints allows researchers to get more eyes on their work and potentially spot weaknesses or errors before formal publication. Versioning can show changes made due to peer feedback. Funders like Wellcome Trust [90] and the National Institutes of Health [91] now allow researchers to list preprints in grant applications and progress reports. Universities should allow researchers to list preprints in evaluation materials and count these as evidence of productivity.

### Personal practice of open scholarship

As described previously, the success of institutional open scholarship initiatives depends in large part on the commitment of individual academics. The best way researchers can support open scholarship is to share their own work. In 2014, at the SPARC open access meeting in Kansas City, I publicly pledged to only edit for, review for, and publish in open access journals [92]. During the years since, I have committed to sharing more products of my research and teaching (Box 5). Other researchers have made similar individual commitments [93–95] or signed on to organized pledges, both as authors (e.g., http://www.openaccesspledge.com and https://moreopenaccess.net) and as reviewers (e.g., https://opennessinitiative.org and [96]). A collection of links to open scholarship pledges can be found via [97].

**Box 5. My open pledge.**

As an open scholar, I pledge to:

1. edit and review only for open access journals,
2. publish only in open access journals,
3. openly share my working manuscripts as preprints,
4. openly share my code and data under version control,
5. openly share my electronic laboratory notebooks,
6. sign my manuscript reviews,
7. preferentially assign openly licensed materials in my classes,
8. create openly licensed teaching materials,
9. ask my professional societies to support open scholarship,
10. speak out in support of open scholarship.

Personal commitments to open scholarship are not made lightly and are often made knowing that many academic environments do not, at present, adequately support such stances. Practicing open scholarship comes with a variety of challenges. The following is not an exhaustive list of these challenges but are some I have faced personally, along with suggestions as to how they could be addressed. I do not believe any of these challenges are insurmountable, but they should be considered if universities want to increase adoption.

#### Economic challenges

**Fig 2:**
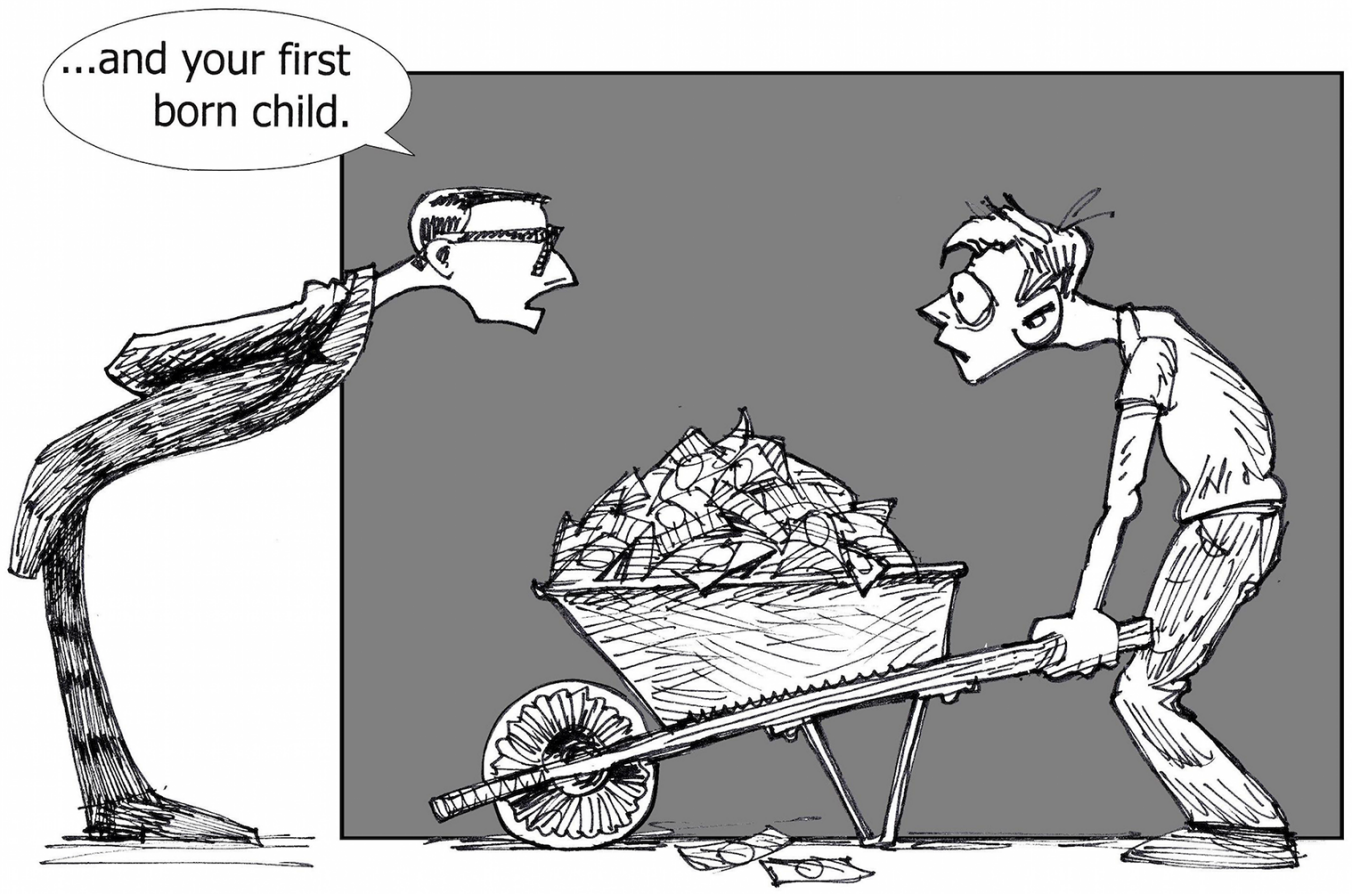
The high cost of publishing. Image: John R. McKiernan and the “Why Open Research?” project (http://whyopenresearch.org).

While free and low-cost open publishing options do exist [6], article processing charges (APCs) for many open access journals are high (Fig 2), with average estimates ranging from about US$900 [98, 99] to about US$1,800 [100], depending on the set of journals studied. Most open access journals provide waivers, but these are typically only automatic for researchers in low income countries. Mexico, where I work, is classified as an upper middle-income country [101], but we have limited funds for research and little to no institutional funds for publishing. When we are offered waivers, they are usually partial—up to 50% off the APC—and the cost is still beyond what we can afford. Because I pledged to publish only in open access journals, publishing in subscription journals and self-archiving is not an option for me. Even if it were, many subscription journals have significant submission, page, and color charges [102]. Thus, for researchers in Mexico and other similar countries, cost is an ever-present consideration and a strong determinant of where researchers choose to publish. Some of the high-profile and more expensive venues are out of our reach, which affects our visibility as researchers. Open access funding models besides “author pays” have to be explored. In Latin America, many journals are free for readers and free for authors, which is possible because of funding from governments, institutions, or cooperative efforts [103]. Universities in other parts of the world should study Latin American journal funding models for guidance and consider how they could support new publishing models for sustainable and affordable open access. The means to finance these new models could come from redirecting journal subscription funds in strategic ways and/or redirecting funds spent on proprietary software licensing, as discussed more below.

#### Technical challenges

Sharing code and data is more complicated than sharing articles, in part because these research products are much more varied, especially across disciplines. In addition, there seems to be less guidance available as to the preferred file formats and organization, the level of documentation needed, different license types, and the best places to archive code and data than there is for articles. Even the most motivated researchers can find navigating these issues frustrating [104]. One standard that most agree on is that code should be shared under version control [105, 106], in which every change is tracked and users can return to previous versions at any time [107], but this is not trivial. Version control tools, like Git, are not always intuitive and most researchers do not receive such training. The barrier to entry is high, and researchers may be reluctant to invest the time needed to become proficient [108]. Or, researchers may be willing to learn but simply be unsure where to start and what resources to use.

Similar challenges arise with open electronic notebooks. Currently, my lab uses Jupyter notebooks [109] to document our research, but this tool requires that students are familiar with both Python and Markdown and also presents a somewhat high barrier to entry, although arguably lower than with raw code alone. Such barriers are particularly relevant when working with undergraduate students, who often receive little to no training in programming or other computer languages. The time involved to learn such tools can be a limiting factor, because these students typically spend only 6 months to a year in my lab and need to hit the ground running. Educational initiatives could address these challenges. Universities could offer courses on essential research skills, including version control and basic programming. These should not just be weekend workshops but courses integrated into all plans of study, beginning at undergraduate and continuing up to graduate levels of education.

#### Redirect funds to address challenges and support academics

I see economic and technical challenges as going hand in hand, with solutions for the latter potentially also providing the means to address the former. Many institutions spend hundreds of thousands to millions of dollars per year on site licenses for proprietary software [110, 111] and continue to invest time and effort in training academics in these closed tools. For example, in 2017, the University of Washington set aside over US$3.6 million for purchasing software licenses [111]. Imagine what amazing things could be done if we redirected even half of that money into supporting open solutions, like open source software and open access publishing.

However, the problems with supporting proprietary software extend beyond just financial costs; there are academic freedom and educational costs as well. As the free software definition outlines, we are less interested in “free as in beer” than we are in “free as in speech” [112]. We want the freedom to run, explore, modify, and redistribute the underlying source code. The use of closed software can leave students and faculty less well equipped, because many analysis functions exist as “black boxes,” in which we can't see, and are rarely forced to understand, what is being done with the data. As Red Hat founder, Bob Young, writes [113],

*Would you buy a car with the hood welded shut?…We demand the ability to open the hood of our cars because it gives us, the consumer, control over the product we've bought and takes it away from the vendor…Having control over the technology they are using is the benefit that is enabling users of open-source tools to build more-reliable, more-customized and lower-cost systems than ever before*.

In the spirit of being smart consumers who retain control over our academic tools as well as the freedom to innovate, I believe universities should shift to open source solutions and provide training in open source alternatives to proprietary software. Data management courses could use LibreOffice Calc instead of Microsoft Excel. Design classes could use GIMP and Inkscape instead of Adobe Photoshop and Illustrator. Programming classes could use primarily Python, rather than Matlab. This latter suggestion would especially help students learn how to design algorithms, write their own functions, and hit the ground running when they get their hands on computational models or data in their final year(s) of study. Training should also include showing students how to give back by contributing to open source projects. In the process of sharing their bug fixes or new functions with the online software community, they would learn good coding practices, version control, and the use of tools like Git. Thus, switching to open source solutions could improve education, thereby addressing some of the technical challenges outlined above.

As an added bonus, many open source programs are also “free as in beer,” or cost much less than proprietary software, typically charging only for things like formal software support. The money saved in student and faculty licenses if universities switched to open solutions could then be redirected to support open innovation or address economic challenges of open publishing. Listed in Box 6 are just a few ideas, which could be scaled depending on institutional resources and needs.

**Box 6. Supporting open source and innovation.**

1. **Develop a 2–5-year plan to move to open source software.** A formal assessment should be conducted to determine which proprietary software products are widely used and which are underutilized by the university. The former could continue to be supported for some time, while the latter would be phased out more quickly. Software for which open source alternatives already exist would be canceled first to liberate funds that could be immediately redirected. Faculty could continue to purchase licenses independently but would not receive institutional support past prearranged cutoff dates.
2. **Offer financial incentives to faculty to develop or improve open source alternatives to proprietary software.** Grants to develop new open source software could be for 1–2 years and offer US$5,000–US$10,000. A few bigger projects might be funded depending on demand and complexity of the software needed. Larger awards would be possible as more software licenses are phased out and more funds liberated. All software development should be done in the open via platforms like GitHub or BitBucket, which could have the advantage of bringing in outside collaborators at no added cost to the university. Smaller grants or faculty prizes could also be awarded for demonstrated contributions to existing open source projects.
3. **Redirect site license funds into supporting open access publishing.** Redirecting funds could also help address economic challenges of open publishing. For example, if a university's site license budget is similar to University of Washington's [111], US$1 million–US$1.5 million (less than half) could be used to set up an institutional open access publishing fund. If universities do not wish to support article processing charges (APCs), they could instead use the funds to support open publishing consortia (e.g., Open Library of Humanities https://www.openlibhums.org) or explore new models.

### Personal practice meets institutional policy

In my view, one of the biggest challenges open scholars face at the institutional level is how they are evaluated for promotion and tenure decisions. There are tensions created by inconsistencies between stated institutional values and evaluations in practice. For example, institutions often emphasize the importance of community engagement and public outreach in their mission and vision statements (e.g., [62, 114, 115]). However, surveys show that faculty feel this support rarely translates into recognition in promotion and tenure. Pretenure faculty report being actively "discouraged" from spending time on community engagement or public outreach activities that take time away from producing “real scholarship,” like peer-reviewed articles [60, 116–118]. Harley et al. conclude that academics who spend significant time on activities like writing for the general public may be "stigmatized for being 'public intellectuals‴ [60].

Similarly, institutions often tout the importance of collaborative and interdisciplinary research (e.g., [119, 120]). Yet, many evaluation systems continue to focus primarily on individual accomplishments, insisting that researchers demonstrate “independence,” and may even include criteria that disadvantage those working in collaborative efforts [60, 121]. For example, some evaluation systems give priority to first or corresponding authorships and devalue middle authorships on publications, especially with larger numbers of authors [122, 123]. The dominance of the journal article over other products as the "basic unit of scholarship" [124] is also a problem lamented by faculty [60, 125]. Surveys report that data, software, online resources, and other digital products are often relegated to “tool development,” given “secondary status,” and may not count at all unless worked somehow into article format [60, 116]. This can be true even when there is interest in and use of the product by academic peers, creating a mismatch between community and institutional recognition [60].

The use of proxy measures, like journal impact factor (IF), to judge the quality and importance of articles is still pervasive in academic evaluations [60, 126] (e.g., [127, 128]), despite studies showing that IF correlates poorly with the scientific quality of individual works [129]. Faculty report feeling intense pressure to publish in specific high IF venues [60, 126, 130]. Institutional requirements may also lead researchers to break apart research projects into smaller, less in-depth units to increase publication numbers [60, 130] or communicate their research in venues that may not reach their ideal audience, just for the sake of prestige [60]. It is understandable that people align their practices with institutional policies related to hiring, promotion, and tenure and with the academic culture in which they find themselves embedded. We, as researchers, want to get, keep, and be successful at our jobs so we can continue doing the work we enjoy. We want recognition from our peers and institution. However, it is not hard to imagine that making decisions that are contrary to what we believe is right or good for our research could create stress, job dissatisfaction, and, in some cases, weaker scholarship. None of these outcomes is good for either faculty or institution.

Those in senior leadership roles at universities can support faculty and promote open scholarship by ensuring that incentives exist to encourage and reward sharing. In the action items listed throughout, I propose several ways that shared code, data, educational resources, outreach activities, preprints, and more could be recognized by committees. These and other suggestions to reform promotion and tenure evaluations are summarized in Box 7. Several of these recommendations arose from discussions among the Advancing Research Communication & Scholarship (ARCS), OpenCon, and SPARC communities (http://bit.ly/PTreform), which include students, postdocs, and pretenure faculty who are understandably concerned about how evaluation criteria will affect their career prospects and advancement. Unfortunately, while early-career researchers (ECRs) may be the best equipped to say how evaluation criteria affect career development or to propose ways of evaluating new forms of digital scholarship, they are rarely given formal opportunities to do so. Senior leadership could support ECRs by giving them more of an institutional voice and including ECR representatives on faculty senates, hiring committees, and tenure review boards.

**Box 7. Recommendations to reform promotion and tenure evaluations.**

1. **Stop using journal-level metrics**, like impact factor, to evaluate the quality and impact of research articles. Institutions can sign the San Francisco Declaration on Research Assessment (http://www.ascb.org/dora).
2. **Use article-level metrics**, such as citation counts, as one quantitative measure of article use and impact. While citation counts are not perfect, they are more representative than journal-level metrics of the impact of individual articles.
3. **Use alternative metrics**, such as tweet activity and media coverage, as one way of evaluating the broader societal impact of research works.
4. **Consider shared code and data** deposited in public repositories as research products that count in evaluations. Quantitative measures of impact could include citations, repository forks, and pull requests.
5. **Consider preprints** as evidence of academic productivity. Preprints do not necessarily have to count as highly as peer-reviewed articles but should still count in evaluations. Support for this perspective comes from the recent Accelerating Science and Publication in biology (ASAPbio) meeting and movement [131].
6. **Value scientific outreach**, such as blogging and articles in popular media, as academic outputs that count in evaluations.
7. **Make forms flexible** by adding space for researchers to describe nontraditional research outputs and their open scholarship activities.

Institutions may take even stronger stances in favor of open scholarship. A policy similar to that at the University of Liège, which requires that faculty upload their work to the institution's open access repository to be considered in promotion and tenure evaluations [132], could be put in place. Of course, for institutions in which the governance structure does not support such a top-down approach, open scholarship initiatives will have to be discussed and agreed upon on at the level of colleges, schools, or even individual departments. Universities can also take guidance from the Leiden Manifesto on research metrics, which includes recommendations for better aligning evaluation criteria with institutional missions, considering disciplinary differences, and taking into account qualitative indicators [133].

### The importance of institutional culture and signals

Reforming evaluations will be a huge step towards more widespread adoption of open scholarship. However, changing policies alone will likely not be enough to transform universities and make sharing the norm rather than the exception. Problems with evaluation systems can be viewed as a symptom of a much bigger problem, namely, an academic culture that has come to favor quantity over quality, labels over content, individual over group accomplishments, and prestige over public good. Universities play a crucial role in determining this cultural environment. Through career advancement decisions, funding and space allocations, faculty prizes, press releases, and even website content, the university signals to academics what it values and what is required to be an accepted member of the community. As in any culture, there is a sense of belonging fostered by what is seen to be a set of shared interests and values. Missions statements are intended to explicitly outline those shared interests and values for the university community, but these words can end up being empty when the institution signals through its actions that its values are different or conflicting. Faculty pay acute attention to these signals and can feel strong pressure to align their practices accordingly. This may be especially true for faculty just starting out, who are working to integrate themselves into their new environment and become valued community members. Thus, "the culture of an institution…is a strong force affecting faculty values and activities" [134].

Importantly, I see the actions I have proposed throughout not so much as a dramatic shift towards new academic cultural values, but more as a return to old ones. Broadening our definition of scholarship, valuing public engagement, wanting the university to be a force for positive social change—these are not new ideas [134–136]. These are old ideas that have taken a back seat to increasingly distorted priorities. I think what universities need is a “realignment” such that what they say they value is better reflected in how they act. University mission statements have to be more than just words.

## Conclusions

I have outlined my vision of a university that endorses the principles of open scholarship, not just in words but in practice, and actively supports faculty in sharing their work. This support can span a continuum from simple steps, like providing space on evaluation forms for faculty to describe their open scholarship or outreach efforts, to more complicated actions, like the redistribution of institutional funds to finance open initiatives. I realize universities may not be able to enact all the reforms I have proposed; some may not be possible due to certain university governance structures, and others may meet with significant resistance. However, if universities work towards just a few of these reforms over the next 2 to 5 years, I think they could significantly increase the adoption of open scholarship practices. The most impactful reforms, as suggested by faculty surveys, are likely to be changes made to evaluation criteria to better recognize and reward diverse types of open scholarship, accompanied by outward signaling from universities that these activities are valued. Such changes may be challenging to enact, but I argue it is worth the effort. As universities embrace sharing, they will likely find it has broad benefits, increasing their visibility, funding, and recruitment power and, most importantly, helping institutions meet core missions like dissemination of knowledge and positive contributions to society.

## Abbreviations

APC: article processing charge
ARCS: Advancing Research Communication & Scholarship
ASAPbio: Accelerating Science and Publication in biology
ECR: early-career researcher
HGP: Human Genome Project
IF: impact factor
MNI: Montreal Neurological Institute
MOOC: massive online open course
OCW: open courseware
OER: open educational resource
SeeSD: Science Education Exchange for Sustainable Development
SPARC: Scholarly Publishing and Academic Resources Coalition
STEM: science, technology, engineering, and mathematics
UNAM: Universidad Nacional Autónoma de México
UNESCO: United Nations Educational, Scientific and Cultural Organization
